# Qualitative and quantitative assessment of Illumina’s forensic STR and SNP kits on MiSeq FGx™

**DOI:** 10.1371/journal.pone.0187932

**Published:** 2017-11-09

**Authors:** Vishakha Sharma, Hoi Yan Chow, Donald Siegel, Elisa Wurmbach

**Affiliations:** Department of Forensic Biology, Office of Chief Medical Examiner, New York, NY, United States of America; University of Helsinki, FINLAND

## Abstract

Massively parallel sequencing (MPS) is a powerful tool transforming DNA analysis in multiple fields ranging from medicine, to environmental science, to evolutionary biology. In forensic applications, MPS offers the ability to significantly increase the discriminatory power of human identification as well as aid in mixture deconvolution. However, before the benefits of any new technology can be employed, a thorough evaluation of its quality, consistency, sensitivity, and specificity must be rigorously evaluated in order to gain a detailed understanding of the technique including sources of error, error rates, and other restrictions/limitations. This extensive study assessed the performance of Illumina’s MiSeq FGx MPS system and ForenSeq™ kit in nine experimental runs including 314 reaction samples. In-depth data analysis evaluated the consequences of different assay conditions on test results. Variables included: sample numbers per run, targets per run, DNA input per sample, and replications. Results are presented as heat maps revealing patterns for each locus. Data analysis focused on read numbers (allele coverage), drop-outs, drop-ins, and sequence analysis. The study revealed that loci with high read numbers performed better and resulted in fewer drop-outs and well balanced heterozygous alleles. Several loci were prone to drop-outs which led to falsely typed homozygotes and therefore to genotype errors. Sequence analysis of allele drop-in typically revealed a single nucleotide change (deletion, insertion, or substitution). Analyses of sequences, no template controls, and spurious alleles suggest no contamination during library preparation, pooling, and sequencing, but indicate that sequencing or PCR errors may have occurred due to DNA polymerase infidelities. Finally, we found utilizing Illumina’s FGx System at recommended conditions does not guarantee 100% outcomes for all samples tested, including the positive control, and required manual editing due to low read numbers and/or allele drop-in. These findings are important for progressing towards implementation of MPS in forensic DNA testing.

## Introduction

Massively Parallel Sequencing (**MPS**) has several advantages over conventional techniques in clinical and forensic applications as MPS can be more cost effective and generates more informative data [1–4]. Clinical applications for genetic diagnoses, risk predictions, and personalized medicine have advanced [5–7]. In forensic testing, MPS will eventually replace current applications of capillary electrophoresis (**CE**), because MPS of short tandem repeats (**STR**s) contributes to a more powerful discrimination based on sequence variants [8] and has the potential to be expanded to include additional targets.

However, before incorporating new technologies into routine laboratory operations they must be evaluated for performance. This may include tests for accuracy, robustness, precision, repeatability, reproducibility, analytical sensitivity, and specificity [1, 6, 9]. In the USA, the Scientific Working Group on DNA Analysis Methods (**SWGDAM**) provides validation guidelines for DNA analysis methods and the National Institute of Standards and Technology (**NIST**) devised the Organization for Scientific Area Committees (**OSAC**) to provide standards and guidelines for forensic science. SWGDAM guidelines have recently been revised to address MPS technologies [10]. Several manufacturers have designed MPS kits for forensic testing that include the Combined DNA Index System (**CODIS**) STRs that are used in the United States for criminal investigations. These kits often include additional autosomal, X, and Y chromosome STRs, as well as identity, ancestry, and phenotypic SNPs (**iSNPs**, **aSNPs**, **pSNPs**). The latter, in combination with complete MPS mitochondrial sequencing kits, could add important information for the identification of missing persons when only partial or skeletal remains are available. These kits were designed to be utilized with the major MPS sequencing platforms currently in use: Illumina’s MiSeq (San Diego, CA) and Thermo Fisher’s Ion PGM and S5 (Waltham, MA).

Recent evaluations of MPS kits and instruments for forensic DNA testing have shown that outcomes are, in general, concordant with CE methods and full profiles could be obtained from as little as 25 to 100 pg DNA. The reports included three HID-Ion AmpliSeq™ Identity Panels (Thermo Fisher Scientific) targeting over 100 identity SNPs (iSNPs) [11–13], two Ion PGM panels (Thermo Fisher Scientific) testing 10 autosomal STRs (**aSTRs**) [14, 15], Promega’s panels testing 17 or 23 STR kits plus amelogenin [16, 17], and Illumina’s MiSeq FGx platform testing their Primer Mix A (153 targets/loci, including 27 aSTRs, 24 Y-STRs, 7 X-STRs, and 94 iSNPs plus amelogenin), and/or Primer Mix B (231 loci, including Primer Mix A plus 22 phenotypic and 56 biogeographical ancestry SNPs) [18–23].

In this study, DNA from 15 individuals (eight males and seven females) was analyzed in detail utilizing Illumina’s MiSeq FGx instrument. In nine experimental runs, over 300 reaction samples were tested. The term “reaction sample” distinguishes the tested samples in an experimental run that were used as replicates, references, or at various DNA inputs from the 15 individual DNAs obtained from volunteers. Reaction samples were tested to evaluate sequencing results based on the number of targets/run (Primer Mix A and B), the number of samples/run (16, 32, and 96), amounts of DNA input/sample and run as well as experimental replication. Analysis focused on the number of reads, which is an equivalent of peak heights in CE. By representing data as heat maps, loci specific read numbers, allele drop-in (**ADI**), and allelic imbalance of heterozygotes can easily be compared for the nine experimental runs. Additionally, detailed sequence analysis of typed and untyped reads, presented here for the first time for Illumina’s MiSeq FGx Forensic Genomics System, revealed true alleles and artifacts. Artifacts included stutter (sequences that are usually one repeat unit shorter or longer than the true alleles) and sequences that showed deletions, insertions, or substitutions of mostly one nucleotide compared to the true alleles. Our findings and discussions may assist future efforts to employ Illumina’s MiSeq FGx system in routine forensic casework.

## Material and methods

### Sample collection, DNA extraction, and quantification

This study was approved by the New York City Department of Health and Mental Hygiene’s Institutional Review Board (IRB# 15–125). Buccal swabs (Citmed Corporation, Citronelle, AL) were obtained with informed consent from 15 volunteers (eight males and seven females). Samples were anonymized. Sequence data was not submitted to a public repository, because this data would allow extracting individual STR profiles and can be used to breach the privacy of the participating volunteers [24]. Furthermore, disclosing individual STR profiles violates New York State Executive Law. All relevant data are within the paper and its Supporting information. Data requests should be directed to Florence Hutner, General Counsel, Office of Chief Medical Examiner (fhutner@ocme.nyc.gov). DNA was extracted using an M48 BioRobot® (Qiagen, Valencia, CA) and the MagAttract® extraction kit (Qiagen) following the manufacturer’s instructions as recently described [18]. Extracted DNA was quantified using Quantifiler® Trio (Thermo Fisher Scientific, Waltham, MA) following the manufacturer’s instructions. A negative control was included with each extraction; if it tested positive, all samples in the batch were discarded.

### Experimental overview and ForenSeq™ DNA signature prep library preparation

This study consisted of nine experimental runs examining only single-source samples. Testing varied five parameters: i) number of targeted amplicons (Primer Mix A: 153 loci and Primer Mix B: 231 loci), ii) number of samples/run (16, 32, and 96), iii) DNA input/sample within a run, iv) DNA input/run, and v) experimental repetition (Table 1). Each experimental run contained reaction samples plus two controls, one positive (Illumina’s 2800M control DNA, always at 1 ng input), and one negative. Library preparation was performed using the ForenSeq™ DNA Signature Prep kit (Illumina, San Diego, CA) following the manufacturer’s instructions. Experimental runs were performed on the Illumina’s MiSeq FGx system in the Forensic mode using the MiSeq FGx Reagent Kit. Illumina’s default settings were specifically chosen for all runs in order to: i) perform fair comparisons between runs and ii) to allow equivalent comparisons between our results and those of other laboratories.

**Table 1:** Overview of experiments. Nine experimental runs were performed with a total of 336 samples: 314 reaction samples, 9 NTCs, and 13 positive controls (2800M). Expt. I is the benchmark.

Experiment Number	Primer Mix^1^	Number of samples	Total DNA per Flow Cell (pg)	Description	Quality Parameters^2^	Q30^3^ (%)	ERN^4^
					A: Cluster density (k/mm^2^)		
					B: Cluster passing filter (%)		
					C: Phasing (%) D: Pre-Phasing (%)		

I	A	32	**31,000**	**Benchmark Run:** 32 samples (including controls), each with 1 ng DNA input as recommended by Illumina for casework samples.	A: **1241**	58.4	1589
					B: 88.9		
					C: 0.186		
					D: 0.065		
II	A	32	31,000	Repeat of Expt. I, using the same kit but 11 weeks later.	A: **668**	48.8	757
					B: 94.91		
					C: 0.213		
					D: 0.031		
III	B	32	31,000	Same samples as in Expt. I but using Primer Mix B (more targets).	A: **613**	57.3	890
					B: 95.78		
					C: 0.157		
					D: 0.086		
IV	A	96	95,000	96 samples (including 5 positive controls), each with 1 ng DNA input.	A: **1471**	48.8	774
					B: 86.18		
					C: 0.139		
					D: 0.102		
V	A	32	10,030	Sensitivity study: 6 samples (3 M and 3 F) at 800, 400, 200, 100, and 50 pg DNA input.	A: **883**	51.5	681
					B: 91.97		
					C: 0.164		
					D: 0.018		
VI	A	32	10,030	Repeat of Expt. V	A: **733**	50.9	506
					B: 94.92		
					C: 0.161		
					D: 0.006		
VII	B	32	10,030	Same samples as in Expt. V but using Primer Mix B (more targets).	A: **913**	47.8	471
					B: 92.9		
					C: 0.171		
					D: 0		
VIII	A	32	16,000	Sensitivity test: same samples as in Expt. I, each with 500 pg DNA input.	A: **614**	47.5	459
					B: 95.85		
					C: 0.166		
					D: 0		
IX	A	16	1,500	Sensitivity test: 16 samples (including controls) with 100 pg DNA input.	A: **893**	39.9	357
					B: 92.4		
					C: 0.132		
					D: 0		

^1^Primer Mix A has 153 loci; Primer Mix B has 231 loci

^2^ Acceptable range for quality parameters—A: Cluster density: 400–1650 k/mm^2^; B: Cluster passing filter: ≥ 80%; C: Phasing: ≤0.25%; D: Pre-Phasing: ≤0.15%.

^3^ Error probability: The percentage of bases that have a quality score >30 (1 base call out of 1000 is predicted to be incorrect) generated after the 25^th^ cycle. The higher the percentage the better the run quality.

^4^ERN: average Experiment Read Number (the average read number for each experimental run calculated by using the true alleles for all samples and loci, including a-, Y-, X-STRs, and iSNPs)

### Data analysis

Primary analysis of MiSeq FGx sequencing data was performed using the default settings of the ForenSeq Universal Analysis Software (**UAS**, Illumina). The UAS report for STRs showed the genotype, flags (i.e. quality indicators for a locus), and coverage information for each locus, which included: allele name, sequence(s), read numbers, and whether or not an allele was typed (i.e. called). A typed STR allele may or may not be correct. Typing indicates that a specific allele sequence reached the interpretation threshold, also referred to as a stochastic threshold. Read numbers above the interpretation threshold are not impacted by stochastic effects and it can be assumed that a drop-out of a sister allele in heterozygous genotypes is unlikely. The interpretation threshold for most loci was >4.5% and the analytical threshold, which distinguishes signal from noise, was >1.5%. Read numbers below 10 were not shown by the UAS software. The UAS report for iSNPs also contained genotypes, flags, and coverage information which included read numbers and whether or not a SNP was typed. UAS “flags”, referred to a specific locus. Flags included: “many alleles” (**ma**); “imbalance” (**i**); “stutter” (**s**); “interpretation threshold” (**it**); “low coverage” (**lc**); “inconclusive” (**INC**), and in addition for iSNPs “noise”. Read numbers below 10 were not displayed by UAS software using default settings. The nine experimental runs tested 314 single-source reaction samples from 15 volunteers and the data for a-, Y-, and X-STRs, as well as the data for the iSNPs was analyzed and compared to the reference data obtained from multiple experiments. Analysis included read numbers of true alleles and artifacts such as stutter and sequence errors. True alleles were alleles that showed the highest read numbers. Sequence errors were sequences that contained sequence deviations (insertions, deletions, or substitutions of nucleotides) when compared to true alleles. Some loci showed more than two typed alleles due to typed stutter and/or allele drop-in (**ADI**), and were subsequently flagged “ma” by the UAS software. iSNPs were typed if their read numbers were >30. If read numbers of one of the alleles was below 30 the locus was flagged “it” and if both alleles were below 30 the locus was flagged “lc”. These untyped reads were included in data analysis.

**Fig 1:**
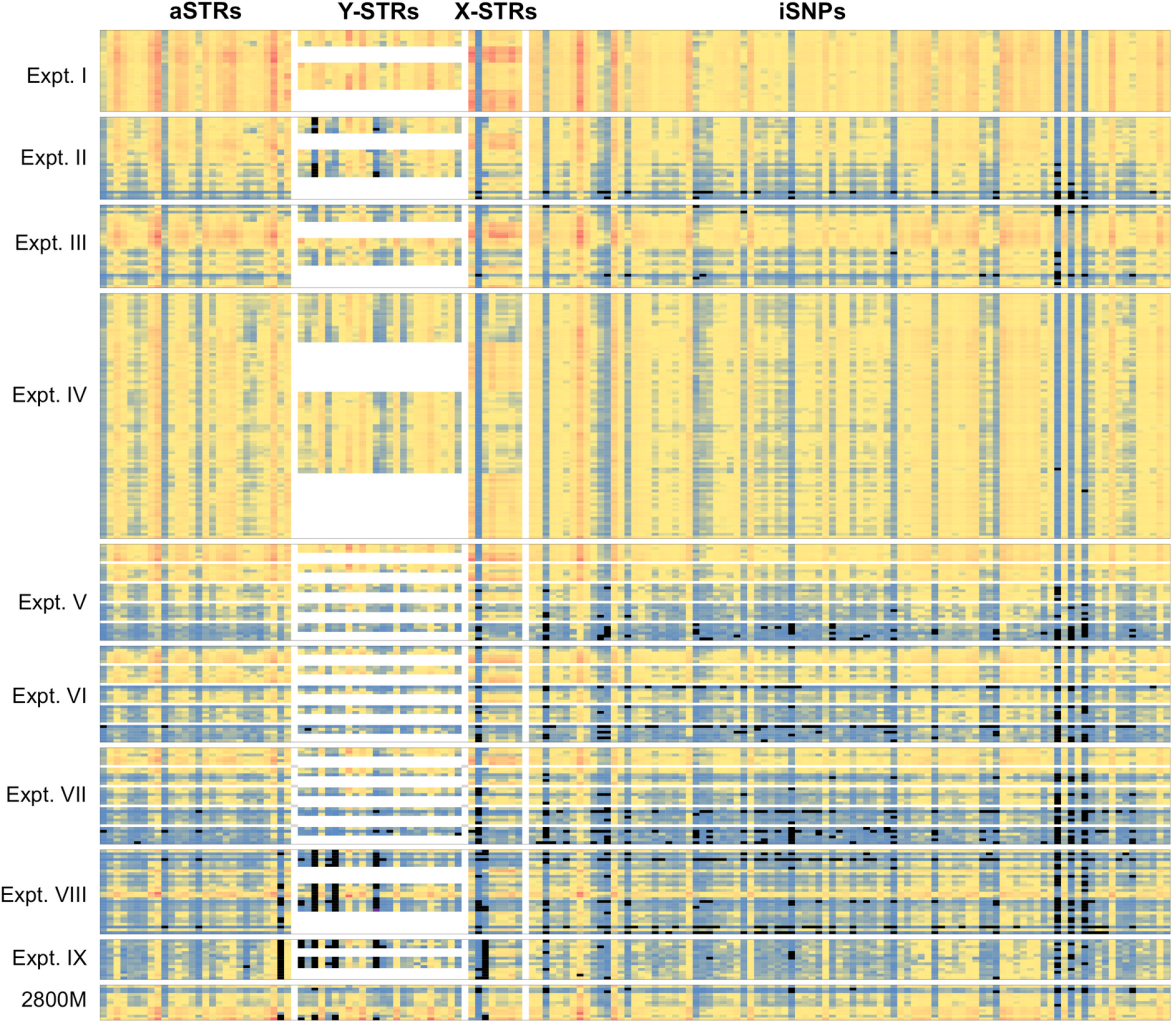
Heat map of read numbers for all samples and loci. The read numbers are shown in color code (low, medium, and high in blue, yellow, and red, respectively) for each sample and locus. Loci shown in columns: **aSTRs (28):** AMEL, TPOX, D3S1358, FGA, D5S818, CSF1PO, D7S820, D8S1179, THO1, vWA, D13S317, D16S539, D18S51,D21S11, D1S1656, D2S441, D2S1338, D4S2408, D6S1043, D9S1122, D10S1248, D12S391, Penta E, D17S1301, D19S433, D20S482, Penta D, D22S1045; **Y-STRs (24):** DYF387S1, DYS19, DYS385a-b, DYS389I, DYS389II, DYS390, DYS391, DYS392, DYS437, DYS438, DYS439, DYS448, DYS460, DYS481, DYS505, DYS522, DYS533, DYS549, DYS570, DYS576, DYS612, DYS635, DYS643, Y-GATA-H4; **X-STRs (7):** DXS10074, DXS10103, DXS10135, DXS7132, DXS7423, DXS8378, HPRTB; **iSNPs (94):** rs1490413, rs560681, rs1294331, rs10495407, rs891700, rs1413212, rs876724, rs1109037, rs993934, rs12997453, rs907100, rs1357617, rs4364205, rs2399332, rs1355366, rs6444724, rs2046361, rs279844, rs6811238, rs1979255, rs717302, rs159606, rs13182883, rs251934, rs338882, rs13218440, rs1336071, rs214955, rs727811, rs6955448, rs917118, rs321198, rs737681, rs763869, rs10092491, rs2056277, rs4606077, rs1015250, rs7041158, rs1463729, rs1360288, rs10776839, rs826472, rs735155, rs3780962, rs740598, rs964681, rs1498553, rs901398, rs10488710, rs2076848, rs2107612, rs2269355, rs2920816, rs2111980, rs10773760, rs1335873, rs1886510, rs1058083, rs354439, rs1454361, rs722290, rs873196, rs4530059, rs1821380, rs8037429, rs1528460, rs729172, rs2342747, rs430046, rs1382387, rs9905977, rs740910, rs938283, rs8078417, rs1493232, rs9951171, rs1736442, rs1024116, rs719366, rs576261, rs1031825, rs445251, rs1005533, rs1523537, rs722098, rs2830795, rs2831700, rs914165, rs221956, rs733164, rs987640, rs2040411, rs1028528. Reaction samples are in rows. Experiments are boxed. Note, female samples did not show any read numbers at the Y-STRs and were kept in white. Locus drop out (LDO) are marked in black. **Positive controls (2800M)** of each experiment are shown at the bottom (kept in the same experimental order as in the figure; for Expt. IV a total of 5 positive controls were included).

**Fig 2:**
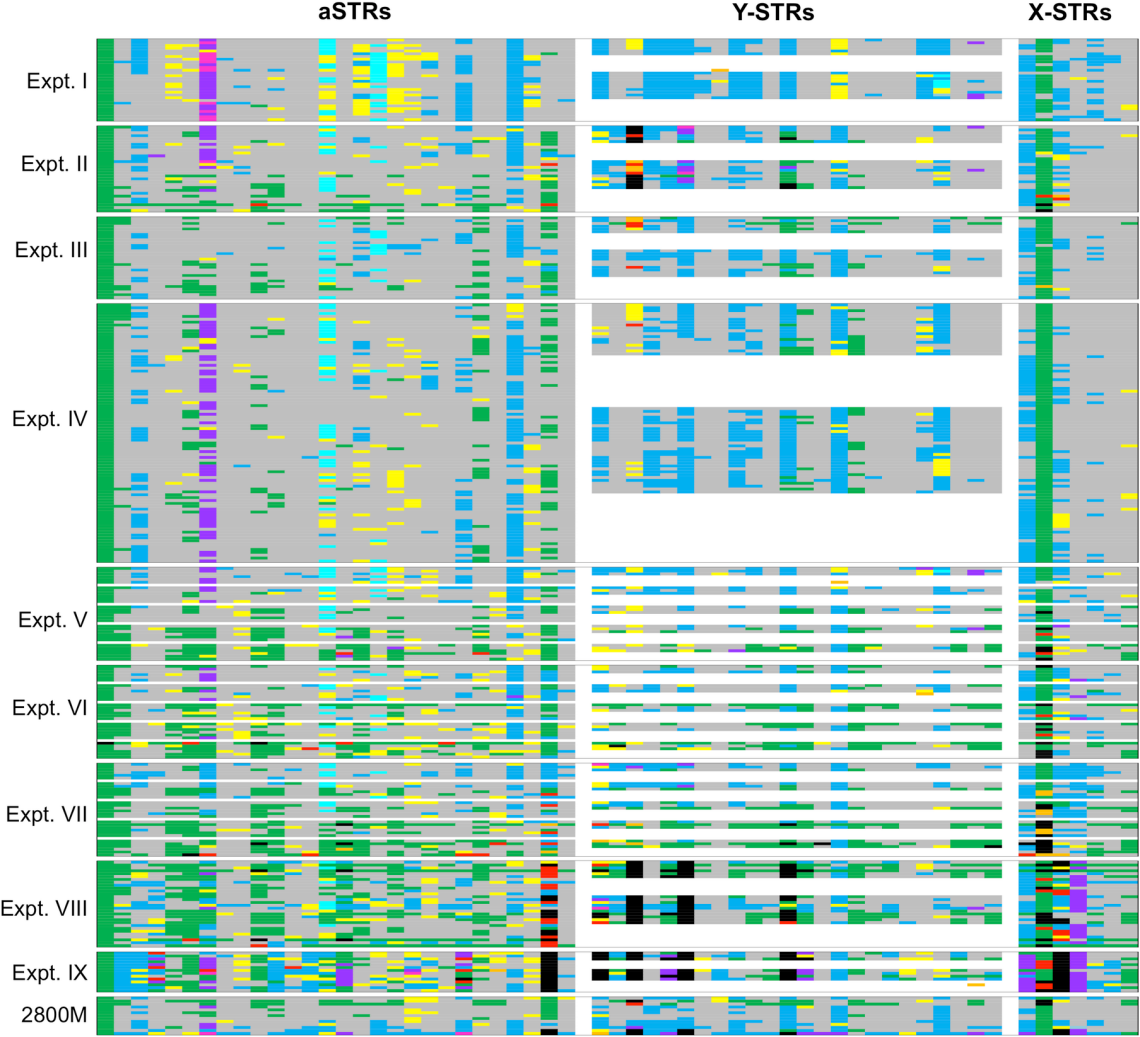
Heat map of STR sequence analysis for all samples and loci. Loci in columns follow the same order as in Fig 1 for aSTRs (28), Y-STRs (24), and X-STRs (7). Reaction samples are in rows. Experiments and positive controls (2800M) are boxed. Color code: **red**–genotype error not flagged by UAS; **orange**–genotype error flagged by UAS; **pink**–typed stutter plus typed sequence error (**SE**); **purple**–typed SE; **yellow**–typed stutter; **black**–locus drop-out (**LDO**); **gray**–untyped stutter; **turquoise**–untyped SE plus untyped SE from stutter; **light blue**–untyped SE; and **green**–no artifacts. Note, female samples did not show sequences at Y-STRs and were kept in **white** (except ADIs, see text). The **white** spacing between a-, Y-, and X-STRs separates the STRs.

**Fig 3:**
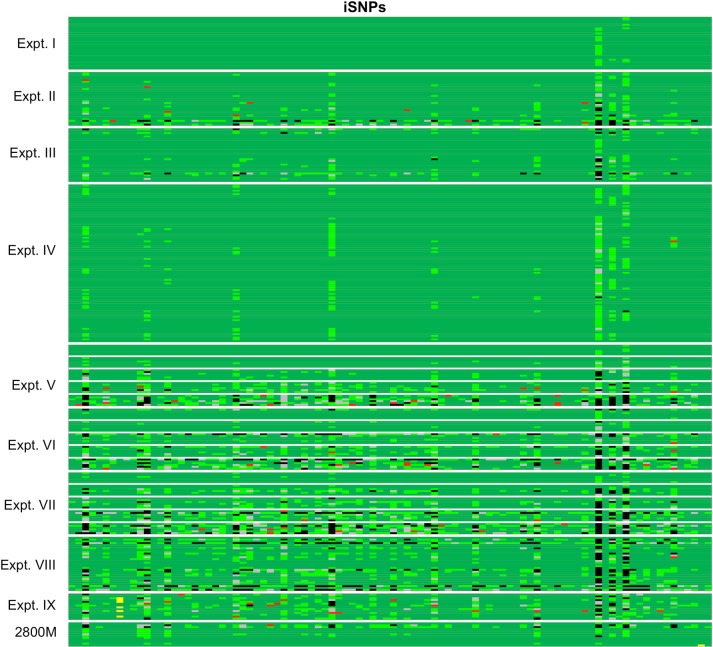
Heat map of iSNP genotype analysis for all samples and loci. Loci in columns follow the same order as in Fig 1 for iSNPs (94). Reaction samples are in rows. Experiments and positive controls (2800M) are boxed. Color code: **green**–correct genotype; **light green**–editable genotype (see text); **yellow**–additional C-allele; **red**–genotype error; **gray**–ADO; **black**–LDO.

**Fig 4:**
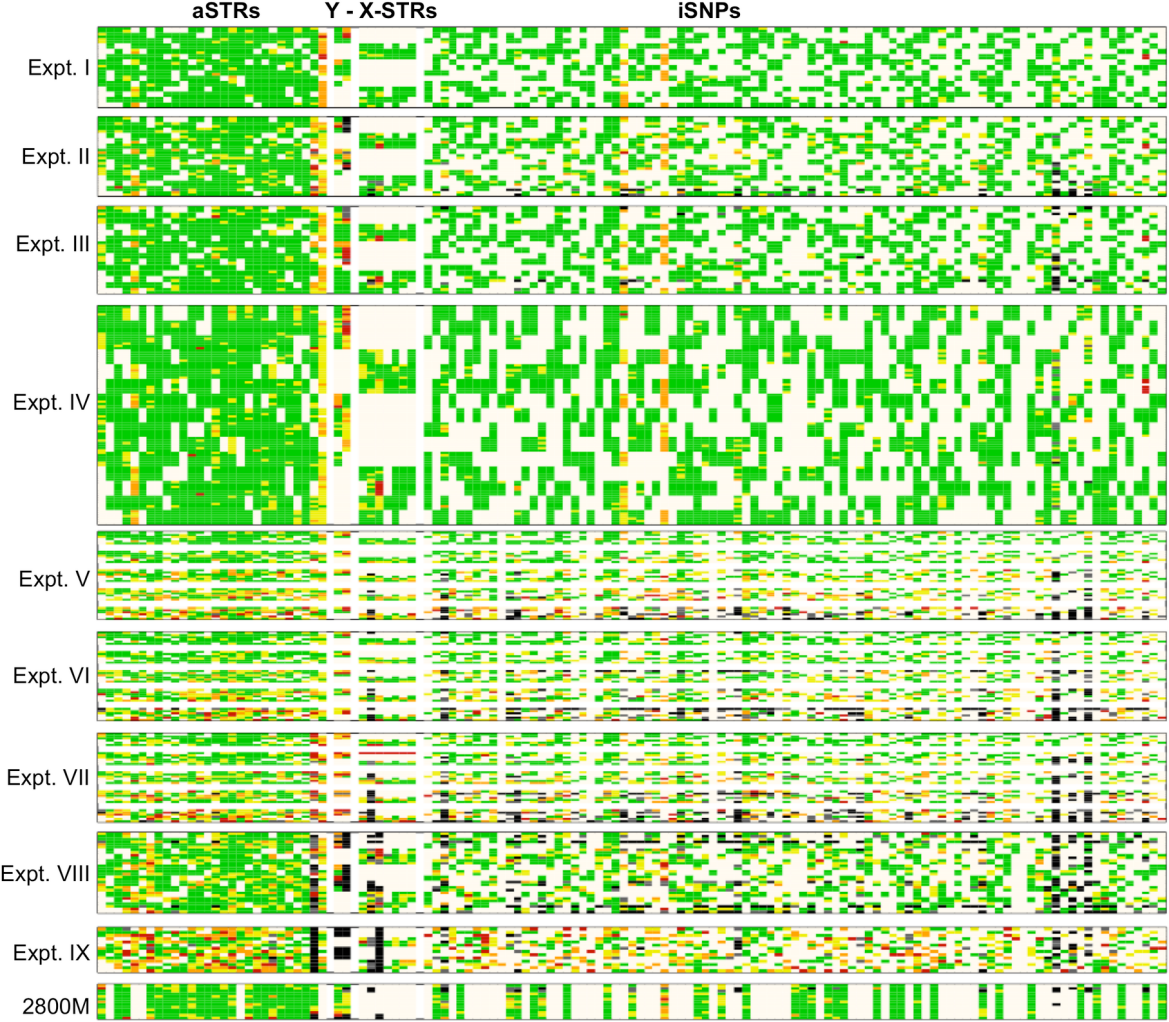
Heat map of ACRs for all samples and loci. Loci in columns follow the same order as in Fig 1 for aSTRs (28), Y-STRs (2): DYF387S1 and DYS385a-b; X-STRs (7), and iSNPs (94). Reaction samples are in rows. Experiments and positive controls (2800M) are boxed. Color code: **green**–ACR ≥0.7; **yellow**–ACR between 0.5–0.7; **orange**–ACR between 0.3–0.5; **red**–ACR ≤0.3; **white**–female samples at Y-STRs, male samples at X-STRs, and homozygotes that did not show an ACR; **gray**–ADO; and **black**–LDO.

Secondary data analysis for generating heat maps and charts (Figs 1–4 and S1–S3 Figs) was performed with Microsoft Excel and R (version 3.3.2) [25].

## Results

### Controls and quality assessment

Using default settings, coverage information of the negative, no template controls (**NTCs**) for all nine experimental runs showed no reads and were consequently flagged “low coverage” for all loci. Positive controls (2800M) were 100% positive in Expt. V–VII. Some iSNPs were missed in Expt. I (3/94) and Expt. IV (2/94) and some STRs were missed in Expt. IX (STR: 5/59; iSNP: 0/94). In addition, some STRs and iSNPs were missed in Expt. III (STR: 3/59; iSNP: 36/94; pSNPs: 9/24; and aSNPs: 25/56), Expt. VIII (STR: 4/59; iSNP: 3/94), and Expt. II (STR: 2/59; iSNP: 28/94). Quality assessment data is shown in Table 1.

### Number of reads per locus

Nine experimental runs were performed in this study. Expt. I was considered the benchmark because it followed the recommendations of the manufacturer for casework samples (32 samples including controls and sample input of 1 ng DNA). The other experimental runs tested more targets (i.e. loci, Primer Mix B), different numbers of samples (16, 32, 96), various DNA inputs (both within and between runs), and replicates (Table 1). In the heat maps, experiment numbers and reaction samples within experiments were plotted by row, while loci were plotted by column. Fig 1 shows the number of reads for true alleles per locus. As may be seen in this figure, overall patterns for specific loci were similar across STRs and iSNPs between different experimental runs, i.e. loci routinely displayed similar read numbers across experiments–loci with high read numbers were consistently high and loci with low read numbers were consistently low.

Average read numbers were determined for each locus. The lowest average read number for aSTRs was 276 [standard deviation (**SD**) 164] for vWA and the highest was 3,593 (SD 2,274) for TH01, revealing a 13-fold difference (Table 2 and S1 Fig). The lowest average read number for Y-STRs was 255 (SD 169) for DYS460 and the highest was 3,386 (SD 2,792) for DYS392, again about a 13-fold difference. Overall, read numbers were slightly lower for X-STRs; the lowest was 68 (SD 53) for DXS10103 and the highest was 3,008 (SD 2,473) for DXS10074, resulting in a 44-fold difference. Generally, read numbers for iSNPs were noticeably lower; the lowest was 34 (SD 16) for rs1736442 and the highest was 1,835 (SD 1,202) for rs1109037, resulting in a 54-fold difference. This indicates that each locus should be considered independently for stochastic and analytical thresholds.

**Table 2:** Sensitivity, dropouts, and ACR.

**Lowest Read Numbers**										
**aSTRs**			**Y-STRs**		**X-STRs**			**iSNPs**		
**Locus**	**Avg read # (SD**^**1**^**)**	**Avg ACR**^**2**^	**Locus**	**Avg read # (SD)**	**Locus**	**Avg read # (SD)**	**Avg ACR**	**Locus**	**Avg read # (SD)**	**Avg ACR**
vWA	276 (164)	0.77	DYS460	255 (169)	DXS10103	68 (53)	0.77	rs1736442	34 (16)	0.78
AMEL	332 (220)	0.72	Y-GATA-H4	295 (182)	HPRTB	1508 (1180)	0.81	rs1031825	46 (22)	0.76
D1S1656	394 (262)	0.76	DYS389II	306 (230)	DXS10135	1711 (1587)	0.63	rs719366	57 (30)	0.79
CSF1PO	590 (371)	0.83	DYS522	342 (233)				rs7041158	65 (39)	0.79
D12S391	696 (492)	0.78	DYS448	426 (470)				rs1294331	67 (37)	0.79
**Highest Read Numbers**										
**aSTRs**			**Y-STRs**		**X-STRs**			**iSNPs**		
**Locus**	**Avg read # (SD)**	**Avg ACR**	**Locus**	**Avg read # (SD)**	**Locus**	**Avg read # (SD)**	**Avg ACR**	**Locus**	**Avg read # (SD)**	**Avg ACR**
THO1	3593 (2274)	0.87	DYS392	3386 (2792)	DXS10074	3008 (2473)	0.78	rs1109037	1835 (1202)	0.82
D20S482	3380 (2233)	0.84	DYS438	3210 (1977)	DXS7132	2509 (2149)	0.85	rs4364205	1293 (864)	0.82
D3S1358	2271 (1497)	0.83	DYS576	3031 (1837)	DXS8378	2344 (2010)	0.78	rs722098	1052 (682)	0.84
D9S1122	2253 (1476)	0.83	DYS505	2331 (1519)	DXS7423	2011 (1640)	0.84	rs430046	948 (610)	0.84
D8S1179	2057 (1319)	0.83	DYS389I	2215 (1267)				rs251934	928 (611)	0.85
								rs2040411	854 (568)	0.85
**Dropouts and Frequency**										
**aSTRs**			**Y-STRs**			**X-STRs**			**iSNPs**	
**Locus**	**ACR**	**f**^**3**^	**Locus**	**ACR**	**f**	**Locus**	**ACR**	**f**	**Locus**	**f**
PentaD	0.67	23/314	DYS385a-b	0.46	33/164	DXS10103	0.77	29/314	rs1736442	85/314
vWA	0.77	4/314	DYS488		23/164	DXS10135	0.63	19/314	rs1031825	52/314
D1S1656	0.76	4/314	DYS390		23/164				rs719366	43/314
AMEL	0.72	2/314	DYF387S1	0.67	10/164				rs129331	37/314
CSF1PO	0.83	1/314	DYS389II		5/164				rs7041158	30/314

^1^SD: Standard deviation

^2^ACR: Allele coverage ratio. The average ACR for aSTRs, X-STRs, and iSNP were 0.79, 0.78, and 0.81, respectively; and the ACRs Y-STRs DYS385a-b and DYF387S1 were 0.46 and 0.67, respectively.

^3^f: Frequency

Interestingly, a-, Y-, and X-STR loci showing the lowest average read numbers were not always the loci with the highest drop-out rate in the nine experimental runs (Table 2). Several loci with higher average read numbers revealed more drop-outs. These were: PentaD, DYS385a-b, DYS390, DYF387S1, and DXS10135. However, for iSNPs, loci with the lowest average read numbers were also those with the largest number of drop-outs (Table 2, lower section). Based on read numbers and drop-out rates, aSTRs performed best, followed by Y-STRs, X-STRs, and iSNPs in that order.

In order to compare different experimental conditions, the read numbers of the true alleles for all samples and all loci (a-, Y-, X-STRs, and iSNPs) tested within an experimental run were averaged to an “experiment read number” (**ERN**, Table 1). This is reasonable within controlled experiments using the same samples because, as previously noted (see above, the findings of Fig 1), while differences between loci within an experiment can vary widely, the relative relationships of loci to each other were consistent between experimental runs (Fig 1 and S1 and S2 Figs). Thus, a comparison of ERN offers some insight into the consequences of changing experimental conditions between runs. Table 3 compares ERNs for a variety of runs testing different conditions. It should be emphasized that due to costs, some of these runs were performed only once. For the comparison of duplicate experimental runs (Table 3/Test 1) the ERNs of Expt. V and Expt. VI were used (Table 1). The ratio of these (ERN of Expt. V: 681 and ERN of Expt. VI: 506) was 1.3, suggesting relatively good repeatability. Replicate runs separated by an 11 week interval (Table 3/Test 2) have an ERN fold-change of 2.1. Reducing reaction sample DNA input by half, from 1 ng to 0.5 ng (Table 3/Test 3), while keeping other variables constant (32 samples and 153 loci, Kit A), resulted in a reduced number of reads by 3.5-fold for the 0.5 ng reaction samples. Increasing sample numbers from 32 to 96 (Table 3/Test 4), while keeping DNA input (1 ng) and the number of loci (Kit A) constant, resulted in a reduced number of reads by about half in the 96 reaction sample experiment. Finally, read numbers also declined when an increased number of loci were sequenced (Kit A, 153 loci vs Kit B 231 loci) when keeping the number of reaction samples and DNA input constant (Table 3/Test 5). Perhaps most important with respect to forensic casework are changes in read numbers when the amounts of DNA were varied within a run. In Expt. V–VII (Table 3/Test 6) sample DNA was present at: 800, 400, 200, 100, and 50 pg. In duplicate runs V and VI (Kit A), the number of reads at 800 pg were 7.4 and 4.5 times greater than those at 50 pg respectively. In Expt. VII, using the same range of sample DNA inputs but with Kit B (increased targets), 800 pg reaction samples had a 6.8 fold increase in read numbers compared to 50 pg samples. This is important in casework where multiple contributors may be present at different concentrations within a sample. It was also observed that female samples within each run had more read numbers at the X-STRs than the male samples (Fig 1 and Table 3/Test 7).

**Table 3:** Effects of experimental conditions on read numbers.

Test	Comparison of Experimental Runs^1^	Fold-Change of Average Read Numbers of Correct Alleles	Results
1. Experimental repeat	V / VI	1.3	Good experimental replication.
2. Kit stability (testing 11 weeks apart)	I / II	2.1	Aged kit appeared to decline in activity.
3. Varying DNA input^2^ between runs—1 ng vs 500 pg	I / VIII	3.5	Reducing DNA input reduced read numbers.
4. Varying the numbers of samples– 32 vs 96	I / IV	2.1	Increasing the number of samples reduced read numbers.
5. Varying the numbers of targets^3^—Primer Mix A vs Primer Mix B	I / III	1.8	Increasing the number of targets reduced read numbers.
	V / VII	1.4	
	VI / VII	1.1	
6. Varying DNA input^2^ within a single run—800, 400, 200, 100, and 50 pg DNA. Read number comparisons only between 800 and 50 pg.	V	7.4	Smaller amounts of DNA within the same run had lower read numbers.
	VI	4.5	
	VII	6.8	
7. X-STRs within a single run—female samples vs male samples	For all Expt.	3.8 vs 1.6	^4^Overall, females with two X chromosomes had higher read numbers than males.

^1^The ERNs of the respective experimental runs were used to calculate the fold-change.

^2^DNA input refers to the amount of sample DNA used at the library preparation stage, not amounts added to the flow cell.

^3^Primer Mix A has 153 loci; Primer Mix B has 231 loci

^4^Compare with Fig 1

### Sequence analysis of STRs

All a-, Y-, and X-STR sequences, whether typed or untyped by the UAS software, were used for data analysis. Because DNA from 15 individuals was repeatedly tested in the nine experimental runs, it was possible to accumulate sufficient data to detect and evaluate sequence inconsistencies such as genotype errors and sequence artifacts. Analysis assessed multiple types of errors including those flagged by Illumina’s UAS software as well as those that were not flagged. Errors evaluated included: sequence errors, stutter, additional alleles, and drop-outs. Fig 2 shows a heat map of a-, Y-, and X-STR loci from all nine experimental runs and the types of errors identified.

### Errors in the STR genotypes

Analysis revealed 79 STR genotype errors of which 71 were not flagged by the UAS software (Fig 2, marked in red and S1 Table). Only eight genotype errors were flagged (Fig 2, marked in orange and S2 Table). Most genotype errors occurred at lower DNA inputs and were allele drop-outs (**ADO**) that resulted in falsely typing heterozygotes as homozygotes (n = 67). Additional genotype errors occurred as a result of ADO plus typed stutter (n = 7, a stutter was considered as a true allele by the UAS software and the resulted genotype varied from the reference sample); locus drop-out (**LDO**) plus typed allele drop-in (**ADI**, n = 1, only the ADI was typed resulting in a genotype that varied from the reference sample, Expt. VIII, 12 reads for allele 7.2 at DYS448, S1 Table); or ADI at Y-STRs in female reaction samples (n = 4, female samples had reads in Y-STRs, which ranged from 13 to 60 at DYS392, DYS505, DYS576, and DYS643, S2 Table). These five ADIs were deemed spurious alleles [26–28] for the following reasons: i) none of the males tested in this study had allele 7.2 at DYS448, ii) allele 16 at DYS576 was not present in any male sample in this experimental run (found in female B5F_400 at 400 pg DNA input in Expt. VI), iii) the two female reaction samples that showed reads at DYS505 and DYS643 had no male reaction samples adjacent on the 96 well-plate during library preparation, and iv) the four female samples showed no other contamination with male DNA. In conclusion, the four ADIs in the female samples were considered as editable. However, at DYS448, the 7.2 ADI with a simultaneous LDO of allele 22 for the male sample B12M_2 was detected only by using reference samples and could not have been detected in an unknown sample. Loci that showed the most STR genotype errors included PentaD (n = 18), DXS10103 (n = 13), DYS385a-b (n = 10), and DXS10135 (n = 6).

In order to assess cross-contamination, two reaction samples, one male and one female, each at 1 ng DNA input, were mixed directly after the PCR reaction that added the sample specific indices (barcoding) and another two reaction samples, same male and female, were mixed after the purification but before the normalization. The resulting sequences of these samples appeared as single-source samples (S3 Table). Additionally, the NTCs of all nine experimental runs were flagged “lc” and did not contain any reads, supporting the notion that pooling samples does not lead to cross-contamination.

### Additional alleles–typed stutter and/or allele drop-in

As shown in Fig 2, some loci were more prone to specific sequence artifacts than others. Loci that showed additional alleles were the result of typed stutter and/or ADI based on typed PCR or sequence errors (Fig 2, marked in pink, purple, and yellow). Sequence errors (**SE**) were defined as sequences that differed from the sequence of true alleles but not due to stutter. In the present study, analysis focused more on SEs than on stutter due to the fact that stutter is a well described artifact in STR amplification (Fig 2, marked in yellow and gray, [29–32]). Within the nine experimental runs, 221 typed SEs were ADIs. Of these, 18 also contained typed stutter, resulting in 3–11 typed alleles per locus (Fig 2 marked in pink and listed in S4 Table). Typed SEs included insertions, deletions, or substitutions (Fig 2, marked in purple and listed in S5 Table). Most typed SEs were found in aSTR D7S820 (n = 117), which at higher DNA inputs revealed insertion of one T at the end of the sequence at rates of <5% to the true allele. At lower DNA inputs, the SEs at D7S820 were single nucleotide substitutions ranging from 22–48% to the true allele. Interestingly, this was not seen in Expt. III and VII when Primer Mix B was used (Fig 2). A second locus that showed many typed SEs was DXS7132 (n = 36). This locus also revealed an insertion of one T. At high DNA input, SEs for this locus were <5% to the true allele, but rose to 14% at low DNA input. Two Y-STRs, DYS643 and DYS390, showed several typed SEs at higher DNA input. The SEs at DYS643 (n = 9) showed deletions of one T at a rate of <4% to the true allele, whereas the SEs at DYS390 (n = 11), showed substitutions of one or two nucleotides. Rates of single nucleotide substitutions were greater than those of two nucleotide substitutions (21–23% vs. 5–9% to the true allele, respectively). At lower DNA inputs, an additional five loci showed SEs: D1S1656 (n = 9), D12S391 (n = 4), DYS389II (n = 4), DYS460 (n = 4), and DXS10074 (n = 11). Most were single nucleotide substitutions, and on rare occasions were two or more substitutions (S5 Table). Regardless, the genotypes for these loci could be determined, and since the SEs showed low read numbers compared to the true alleles, they could be manually edited. In the case of substitutions where STR length did not change, genotyping was not affected.

### Untyped sequence errors

Untyped SEs were those which were not called by the UAS software. These occurred on an average of approximately <10% to the true allele. Analysis of these SEs found that many were of the same kind as observed for typed SEs and were predominantly substitutions. D2S1338, D21S11, and DYS612 showed only single nucleotide substitutions as SEs that were derived from their true alleles as well as from their stutter products (Fig 2, marked in turquoise). These occurred at lower frequencies for D2S1338 and D21S11 (one to six times per run), and more often for DYS612 (up to 16 times per run).

Untyped SEs derived from true alleles were found mostly at the following STRs (Fig 2, marked in light blue): D19S433 (S6 Table), D12S391, D3S1358, DYF387S1, DYS389I, DYS390, DYS437, DYS448, DYS612, DYS438, DYS576, DYS389II, DXS10074, DXS10135, and DXS7423. Sequence analysis revealed single nucleotide substitutions. A few reads also showed substitutions of two and less frequently of three or more nucleotides at DYF387S1 and DXS10074.

### Analysis of iSNP genotypes

iSNP reports include read numbers for all genotypes detected. All iSNP reads, including low reads that were untyped, were used for evaluating genotypes. Two types of errors were identified: i) those which were flagged but could be interpreted (edited), and ii) those which were not flagged and thus, resulted in genotype miscalling. Some errors appeared locus specific as may be seen in columns with numerous errors within and between experimental runs (Fig 3). LDO were also observed.

In the nine experimental runs, 73 iSNP errors were found. As was found with STRs, ADOs led to falsely typed homozygous genotypes (Fig 3, marked in red) that were not flagged by the UAS software. These errors affected 41 iSNPs (loci) at frequencies of six or less. The iSNPs containing the most genotype errors were rs914165 (n = 6), rs6955448 (n = 4), rs9905977 (n = 4), and rs1493232 (n = 4). Interestingly, loci rs914165 and rs6955448 also showed allelic imbalance in heterozygous samples (samples were flagged accordingly, S3 Fig). Similarly, locus rs338882, which revealed two genotype errors, was also prone to allelic imbalance. It is not surprising that loci with allelic imbalance would also be subject to ADO. As with the STRs, the genotype errors for the iSNPs occurred more often at low DNA input (Expt. V–VII, and IX).

Overall, iSNP loci showed a broad range of read numbers (Table 2). As expected, loci with the lowest read numbers had the most LDOs and ADOs (Fig 3, marked in gray and black and Table 2). These were most common at loci: rs1736442, rs1031825, rs719366, rs1294331, rs7041158, rs1357617, rs2920816, rs338882, and rs2342747. Predictably, at low DNA input the occurrence of LDO and ADO increased for these iSNPs (Expt. V–IX).

Genotypes marked with “it” were most frequently typed falsely as homozygotes. However, by considering all reads, heterozygous genotypes could be determined which were in agreement with reference samples. Full read analysis also revealed that inconclusive genotypes (INC), flagged “lc”, showed reads for both alleles (heterozygotes) and were always in agreement with reference samples (S4 Fig). However, if ADO occurred, false homozygote status would be a consequence in a heterozygous sample. As shown in Fig 3, even for experiments performed at recommended conditions as well as using Illumina’s 2800M positive control DNA (Expt. I–IV), many genotypes could only be restored by manual editing (Fig 3, marked in light green). However, these were controlled and not unknown samples.

Only in Expt. IX (Table 1), additional C-alleles leading to three alleles in the bi-allelic iSNPs (A/G, Fig 3, marked in yellow) were found. This occurred in six of the 14 reaction samples at one locus (rs1109037). A second locus (rs2040411) also showed an additional C-allele, but only in 2800M. Five of the six reaction samples were homozygous (A/A) at rs1109037 and one was heterozygous (A/G). 2800M was homozygous at rs2040411 (A/A). The additional C-alleles were <5% of the true alleles. UAS software correctly typed all samples and 2800M. Interestingly, the five homozygous samples at rs1109037 were flagged with “noise”, while the sixth, the heterozygous sample, at rs1109037 plus 2800M at rs2040411 had no flag (Fig 3 and S3 Fig). It is also worth noting that these iSNPs were among those with the highest average read numbers (Table 2).

### STR & iSNP Allele coverage ratio (ACR)

Allele coverage ratio (**ACR**) was determined for heterozygote loci by dividing the lower number of reads by the higher number of reads. Equal numbers of reads result in ratios of one. Increasing imbalance results in smaller ratios. Fig 4 shows a heat map of ACRs for all samples and loci. Overall, two specific ACR patterns were observed: i) specific loci across all runs, regardless of run conditions, showed significant allelic imbalance; and ii) decreasing DNA input and read numbers correlated with increasing allelic imbalance.

The four aSTR loci that had the largest imbalances were: D22S1045, PentaD, D5S818, and AMEL with calculated average ACRs of 0.58, 0.67, 0.72, and 0.72 respectively. Two Y-STRs, DYS385a-b and DYF387S1 (these loci have undergone duplication), also had allelic imbalances and showed average ACRs of 0.46 and 0.67 respectively. Two X-STRs, DXS10135 and DXS10103, showed some drop-outs and their calculated ACRs were 0.63 and 0.77, respectively. Three iSNPs had low ACRs; these were rs6955448 (0.43), rs338882 (0.55) and rs914165 (0.71). However, some of the iSNPs showed none or only a few heterozygous samples (rs938283, rs12997453, and rs2056277) and were excluded from the analysis since an ACR could not be calculated.

The second type of low ACR pattern observed revealed a correlation between reducing DNA input and declining ACRs (Expt. V–VII, and IX). In addition, the read numbers of loci within reaction samples correlated with ACRs (Table 2): i) for aSTRs, the average ACR was 0.79 and four of the five loci with the lowest average read numbers had ACRs below it, while the loci with the highest average read numbers had ACRs that were higher. ii) For X-STRs, the average ACR was 0.78 and one of the two loci that had a lower ACR had low read numbers, but both show allelic imbalance in all runs regardless of run conditions. The remaining loci had higher ACRs. iii) Finally, for the iSNPs, the average ACR was 0.81 and the loci with the lowest average read numbers had ACRs that were below, while the loci with the highest read numbers had ACRs that were above.

## Discussion

It is important that applied tests are evaluated prior to regular use in order to determine their accuracy and limitations. This is crucial for diagnostic tests and clinical screens in order to avoid improper patient treatments [33, 34], as well as for forensic tests to avoid errors in evidence used against defendants [35]. This study focused on a novel method for forensic DNA analysis, massively parallel sequencing (**MPS**). Illumina’s MPS platform, the MiSeq FGx Forensic Genomic System, was evaluated using single-source DNA samples to determine its capabilities and limitations. All runs were performed using the manufacturer’s default settings to make uniform comparisons between runs and to allow other investigators to compare data across laboratories. Recently, this platform was evaluated for concordance, reproducibility, sensitivity, mixed DNA samples, and allele coverage ratio (**ACR**) [18–23]. This study however, focused on read numbers and showed their importance with regard to ACR, ADO, and genotype errors. In addition, a thorough sequence analysis on typed and untyped sequences was performed, which is crucial for a better understanding of the platform. As an aid to data interpretation, outcomes of all nine experimental runs, including 314 reaction samples plus 13 positive controls (2800M) were presented as heat maps. This visualization of the data allows recognition of loci specific patterns of read numbers, ADIs, and ACRs.

### Read numbers

This study showed that relatively high read numbers correlated with good results, i.e. no allelic imbalance (high ACRs) and no drop-outs. However, within reaction samples, read numbers for the various loci differed tremendously. The average read numbers varied between locus-to-locus for aSTRs and Y-STRs over 10-fold, and for X-STRs and iSNPs over 40-fold. Similar data was reported from other laboratories [19, 21, 22]. Illumina’s UAS software for most STRs, including all aSTRs, used default settings of >1.5% analytical and >4.5% interpretation thresholds, which can be adjusted for each locus. This is different from the current CE techniques where measured relative fluorescence unit (RFU) intensity does not differ significantly between loci and therefore the same thresholds are often used for all loci [36, 37].

For iSNPs, loci with the lowest read numbers also showed the greatest ADOs (Table 2). This correlation is not an unexpected finding. However, some a-, Y-, and X-STRs (PentaD, DYS385a-b, DYS390, DYF387S1, and DXS10135) with relatively high average read numbers, but low ACRs (Table 2 and Fig 4), also showed high frequencies of drop-outs across the nine experiments. The nine STRs that showed the highest drop-out frequencies (vWA, PentaD, D1S1656, CSF1PO, DYS385a-b, DYS448, DYF387S1, DXS10103, and DXS10135) also accounted for most genotype errors (57/79, 75%) due to false typing as homozygotes (S1 and S2 Tables). Drop-outs of DXS10103 were also reported previously [20, 23]. This data suggest better balancing of the multiplex amplification reactions may be required.

This study showed that drop-outs occurred in several runs that were performed according to the manufacturer’s instructions (Expt. II–IV, Fig 1) as well as in positive controls (2800M). Therefore, the positive control failed to test all loci within the multiplex kit (Fig 1), meaning it behaved as a reaction sample. The affected loci were predominantly iSNPs, and Y- and X-STRs with low read numbers.

Besides the locus-to-locus variation of read numbers within the multiplex reaction, it was shown that read numbers correlate with the DNA input. Low DNA input led to low read numbers (Table 2) and therefore resulted in higher frequencies of ADO/LDO, low ACRs, and genotype errors (Expt. V–VII, Figs 1–4). Nevertheless, occasionally, full profiles could be obtained from samples with as little as 50 pg DNA [18, 20].

Repeatability was demonstrated with the experimental runs V and VI (Figs 1–4, Table 3), which were performed within a short time period. On the other hand, repeated experimental runs performed 11 weeks apart (Expt. I and II, unexpired kit) showed declined activity: lower read numbers (Table 3 and Fig 1), more drop-outs (Fig 1), more genotype errors (Figs 2 and 3), and lower ACRs (Fig 4). The quality assessment data for Expt. II revealed lower cluster density and a lower Q30 (Table 1). It is unclear why the run performances differed so dramatically. The difference of nearly three months between kit usage might be a possibility (kit aging), but certainly is not confirmed by two experiments. Another confounding factor could be that the MiSeq instrument was serviced (i.e. the fluidics and optics system were calibrated) immediately before Expt. II was conducted, but this was not the case for Expt. I. These inconsistencies demonstrate the need for additional assessments in order to achieve more reliable outcomes.

### Sequence analysis

This study included a detailed analysis of sequence differences within loci in order to learn more about PCR and/or sequencing errors (**SE**). During the MPS process there are several amplifications where a polymerase may incorporate an incorrect base that could result in a noteworthy number of reads: first, during PCR library preparation and second, during clonal amplification on the flow cell. All reads were evaluated–above and below thresholds, called and not called–to detect sequencing errors and to characterize single-source samples in detail for Illumina’s MiSeq FGx™ system. In order to detect genotype errors, 15 individual samples were repeatedly sequenced and were used as reference. Within the nine experimental runs, 152 genotype errors were found, 79 in STRs and 73 in iSNPs. Most of the STR (67/79, 85%) as well as all of the iSNP genotype errors were due to ADO resulting in falsely typed homozygotes. The remaining 12 STR genotype errors occurred because of ADO plus typed stutter (n = 7), LDO plus ADI (n = 1), and ADI at Y-STRs in female samples (n = 4). The majority of genotype errors occurred at lower DNA input. However, it should be noted that a few were also detected in experimental runs that were performed following the manufacturer’s recommended DNA input of 1 ng (Figs 2 and 3, Expt. II–IV), as well as in the positive controls (2800M).

Typed ADI and typed stutter resulting in additional alleles, which reached up to 11 in this study, occurred in each experimental run and needed to be edited by an analyst. This requires additional time and may cause ambiguities (Fig 2 and S3 Fig). This study also focused on the artifacts of PCR amplification that could be observed by sequencing STRs. These artifacts could reach 50% of the true allele in low DNA input samples and fell into two categories: typed and untyped (S5 Table). Typed ADIs were found to be insertions, deletions, or substitutions, which were observed mostly at D7S820, DYS390, DYS643, and DXS7132. These typed ADIs accounted for 172/221 (78%) of the typed SEs and SE plus stutter (S4 and S5 Tables). At higher DNA input, the ADIs were low, approximately 3–5% to the true allele. At low DNA input, ADIs were predominantly substitutions of single nucleotides. Less frequently, two nucleotide substitutions and rarely, three or more nucleotide substitutions were observed. These substitutions could rise to approximately 50% of the true allele. The genotype could still be determined since lengths of the alleles were not affected. These artifacts were considered as editable for determining the genotype [18], but not for the corresponding sequence.

Untyped SEs were commonly found at D2S1338, D21S11, D19S433, D12S391, D3S1358, as well as in several Y-STRs including DYS612, and a few X-STRs (Fig 2). The untyped SEs occurred on average approximately <10% to the true allele. Sequence analysis revealed predominantly substitutions of one, and less frequently, of two or more nucleotides (as observed for the typed SEs). The aforementioned aSTRs contain compound/complex repeat units [8] and another study found similar artifacts in D3S1358 as well as D19S433 [38]. Interestingly, on the ion torrent PGM platform, these two STRs also produced similar artifacts [15]. More analysis will be needed to distinguish the cause for these SEs. Do they occur because of the sequence of the repeat units or due to the design of the multiplex reaction? It is also unclear why the UAS software typed some ADIs and not others since their percentages to the true allele were similar.

It is known that STR amplification is accompanied with by-products such as stutter [32]. Stutter can lead to more than two typed alleles per locus (Fig 2). Stutter is most frequently one repeat unit shorter than the true allele, but some are two or more units shorter or longer [32]. These were also observed in our study (S4 Table). A few genotype errors (7/79, 9%), were due to ADO plus typed stutter (S1 and S2 Tables). In some cases where STRs showed complex repeat patterns, it was possible to obtain more than one stutter product in which different repeat units were missing [e.g. Expt. I D12S391: true allele: (AGAT)11 (AGAC)7 AGAT; stutter A: (AGAT)11 (AGAC)6 AGAT; stutter B: (AGAT)10 (AGAC)7 AGAT]. Sequencing allowed allocation of stutter products to the sequence of a true allele.

The sequencing error rate of the Illumina platform is about 10^−2^ to 10^−3^ (1 nucleotide in 100 to 1,000 bases) [2, 39]. The most frequent errors are single nucleotide substitutions, arising from errors during amplification and sequencing due to polymerase mistakes as well as bases being called incorrectly by the UAS software [39]. The quality score Q30 is given for each experimental run after the 25^th^ cycle and refers to the error probability of 0.001 (Table 1). In addition to chemistry and instrument errors, DNA dependent DNA polymerases may also introduce errors during PCR and sequencing reactions, particularly at STRs, that are difficult to replicate, which is also reflected by the higher mutation rates of STRs compared to other genetic markers during cell division [40–42].

An important issue that must be addressed before the implementation of MPS technologies in both forensics as well as in clinical applications is that of sample mixing or cross-contamination. Illumina’s MiSeq FGx system, as an MPS technology, includes individual sample barcoding (indexing) prior to the pooling of samples for sequencing. Since barcoding with specific primers and subsequent cleanup are performed in separate tubes, this procedure likely is no cause for sample/barcode mix-up. Nevertheless, all evidence in support of this supposition should be published. In this study, cross-contamination was assessed by testing reaction samples that were mixed directly after barcoding (still containing DNA polymerase, dNTPs, and primers) as well as after purification, but before normalization. These samples did not show any sign of DNA mixture (S3 Table). In addition, the NTCs of all nine experimental runs showed no reads. Furthermore, within the 327 reaction samples, including 13 positive controls (2800M), only five ADIs that were deemed spurious alleles were detected at Y-STR loci with read numbers ranging from 12 to 60. Four of these five ADIs were in female samples and one was in a male sample (S1 and S2 Tables). Two of these four ADIs were not present in the experimental setup (library preparation), including one that wasn’t even detected among the 15 individuals used for this study. Regarding the other two ADIs, the two female samples were not adjacent to male samples during library preparation. Since all four female samples showed no further contamination with male DNA these ADIs were considered editable. Similar apparent ADIs in female samples were also observed in another study [22]. On the other hand, the last ADI, which was found in a male sample, was a genotype error that could not have been detected without a reference sample. Taken together, these findings suggest that observed sequence errors were the result of DNA polymerase infidelity rather than contamination during the pooling that is part of MPS.

Again, all reads were considered for iSNP data analysis and often allowed for correct genotyping after editing. iSNP loci flagged “it” could only be called correctly when sub-threshold reads were analyzed. iSNPs flagged “lc” could also be interpreted correctly if they were heterozygotes. However, when analyzing sub-threshold reads that appear homozygous, false genotypes were called in cases of ADO. As expected, the loci showing the lowest read numbers were the ones that needed the most editing (compare Fig 1 with Fig 3). It should be pointed out first, that no wrong alleles were detected, besides the five spurious alleles and the C-alleles. Second, this could only be detected by testing reaction samples repeatedly and comparing the resulting genotypes, which would not be possible with unknown samples. And third, “it” and “lc” flags appeared frequently in experimental runs that were performed following the manufacturer’s instructions, as well as in the positive controls (2800M, Expt. I–IV, S3 Fig). This data suggests a re-evaluation of the iSNPs should be considered before implementation in forensic casework.

## Conclusions

STRs and iSNPs from reaction samples with high DNA inputs had higher read numbers which led to more reliable results: fewer ADO/LDO and higher ACR. Therefore, the DNA input had the strongest effect on the outcome. Consequently, Illumina’s ForenSeq DNA Signature Prep Kit Primer Mix A would benefit from a re-designing of primer and amplification conditions to achieve a more balanced outcome in terms of read numbers.

Genotype errors occurred primarily due to ADO/LDO and were only detected since samples were run repeatedly.

Essentially, no errors were found due to contamination caused by pooling or handling of the samples during library preparation. Spurious alleles were found on a few occasions: typed Y-alleles in four female samples (13–60 reads) that could be easily edited as well as, one ADI in a male sample at DYS448 (Expt. VIII: 500pg, 12 reads) that was only found by comparing the genotype to repeated runs.

Running Illumina’s FGx Forensic Genomic System under ideal conditions does not assure 100% outcomes neither for the reaction samples nor the positive control due to drop-outs and genotype errors. In addition, typed ADI and stutter were observed in all runs and required manual editing.

## Supporting information

S1 Fig Average read numbers of true alleles for aSTRs of all nine experimental runs. (PDF)

S2 Fig ERN of true alleles for aSTRs. (PDF)

S3 Fig Heat map of UAS quality indicators. (PDF)

S4 Fig UAS data output for iSNPs. (PDF)

S1 Table Genotype errors not flagged, Fig 2 marked in red (n = 71). Typed genotype differs from reference sample.
(PDF)

S2 Table Genotype errors flagged, Fig 2 marked in orange (n = 8). (PDF)

S3 Table Assessing cross-contamination by mixing samples directly after indexing and after PCR purification. (PDF)

S4 Table Typed stutter and typed sequence error, Fig 2 marked in pink (n = 18). (PDF)

S5 Table Typed sequence errors, Fig 2 marked in purple (n = 203). (PDF)

S6 Table Untyped sequence errors at D19S433 from one reaction sample of Expt. I. (PDF)
