# Supplementary material for: Qualitative and quantitative assessment of Illumina’s forensic STR and SNP kits on MiSeq FGx™
Source: PLoS One. 2017 Nov 9;12(11):e0187932. doi: 10.1371/journal.pone.0187932 (PMC5679668; doi:10.1371/journal.pone.0187932)
Supplement: S1 Fig — (PDF) [file pone.0187932.s001.pdf]

Suppl. Fig. 1

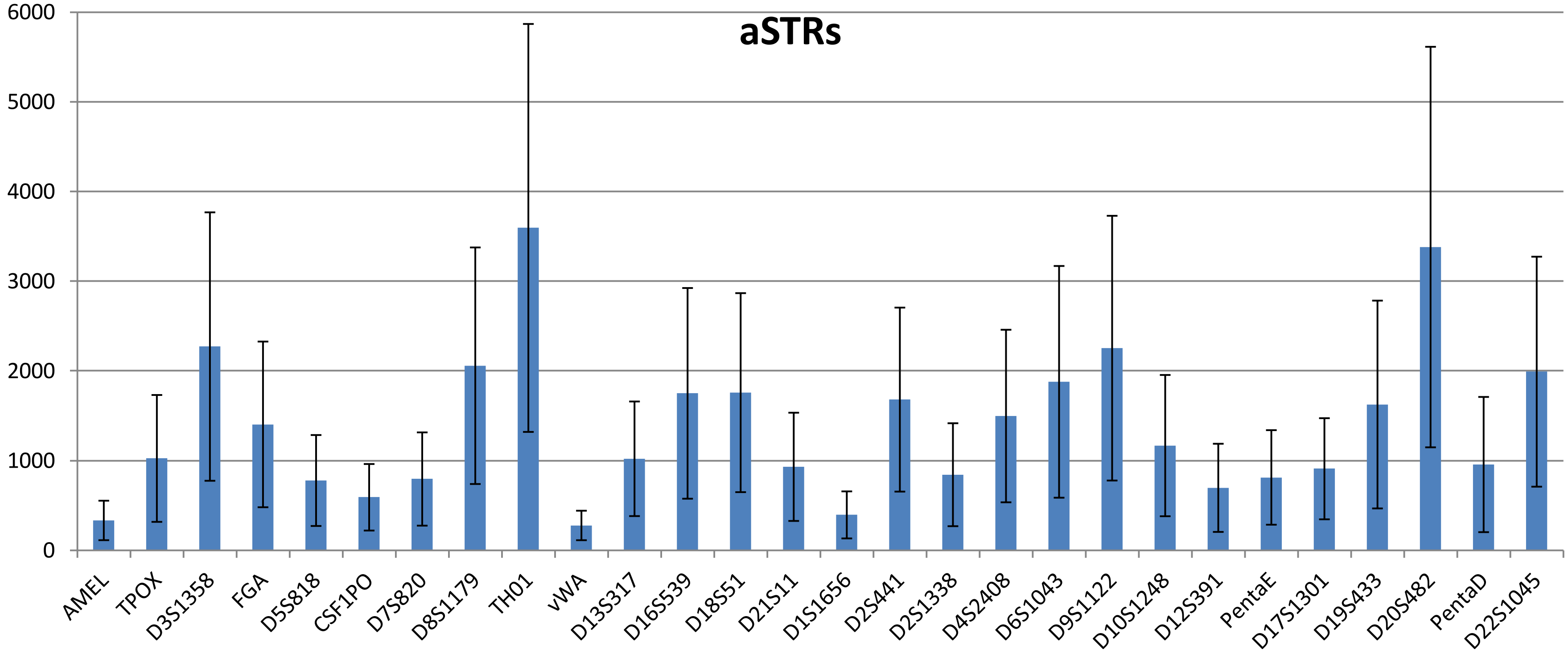

Average read numbers of true alleles for aSTRs of all nine experimental runs:

Error bars show plus and minus standard deviation

X-axis: AMEL and aSTRs; Y-axis: read numbers
