# Supplementary material for: Qualitative and quantitative assessment of Illumina’s forensic STR and SNP kits on MiSeq FGx™
Source: PLoS One. 2017 Nov 9;12(11):e0187932. doi: 10.1371/journal.pone.0187932 (PMC5679668; doi:10.1371/journal.pone.0187932)
Supplement: S2 Fig — (PDF) [file pone.0187932.s002.pdf]

Suppl. Fig. 2

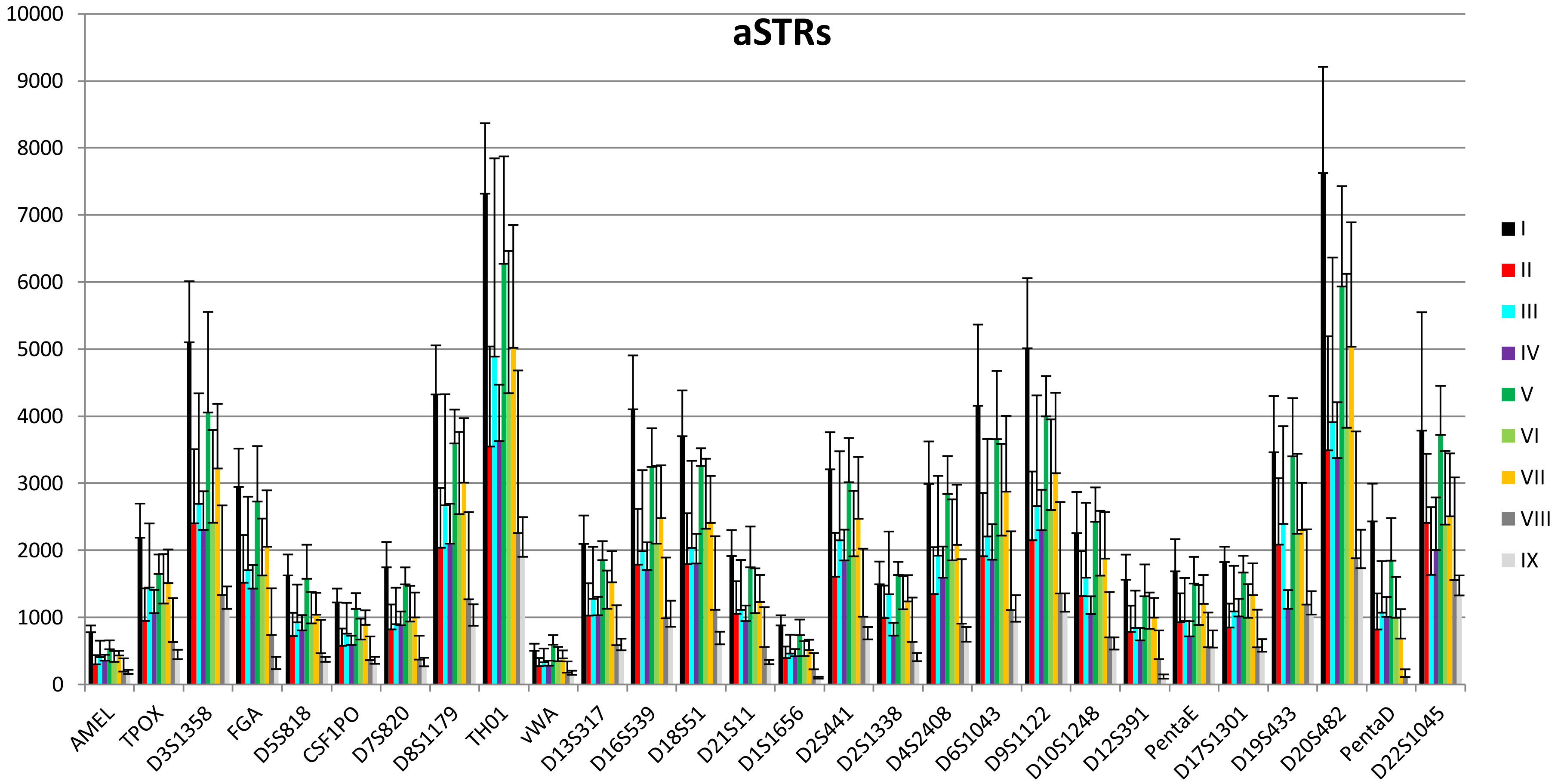

Read numbers of true alleles for aSTRs:

The read numbers (Y-axis) for all samples (Expt. I – IV, VIII and IX) and for the six samples with the DNA input of 800 pg (Expt. V – VII) were averaged for the indicated loci (X-axis). The error bars show the standard deviation. Note, the experimental runs vary in number of targeted amplicons, numbers of samples per run, DNA input, and numbers of samples, only Expt. V and VI are repeated runs.
