# Supplementary material for: Qualitative and quantitative assessment of Illumina’s forensic STR and SNP kits on MiSeq FGx™
Source: PLoS One. 2017 Nov 9;12(11):e0187932. doi: 10.1371/journal.pone.0187932 (PMC5679668; doi:10.1371/journal.pone.0187932)
Supplement: S3 Fig — (PDF) [file pone.0187932.s003.pdf]

Suppl. Fig. 3

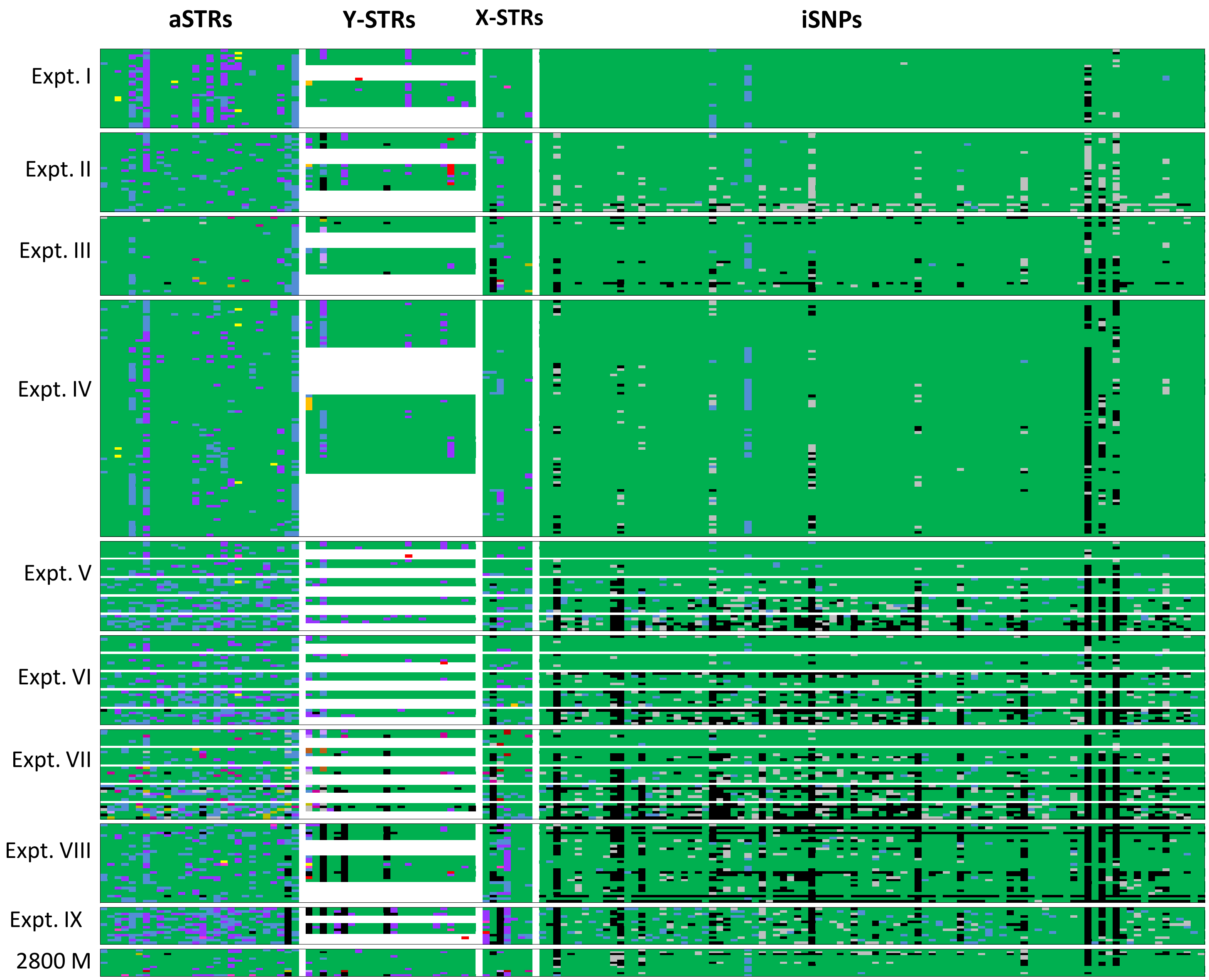

Heat map of UAS quality indicators for all samples and loci:

Samples and loci are organized as in Fig. 1. **Color code**: **red** – many alleles (ma); **purple** – ma and imbalance (i); **dark red** – ma and interpretation threshold (it); **dark pink** – ma, it, and stutter (s); **pink** – ma, i, and s; **light purple** – ma, it, i, and s; **yellow** – s; **orange** – s and i; **khaki** – s and it; **black** – low coverage (lc); **gray** – it; **blue** – i; **green** – no flag . Note, female samples did not show sequences at Y-STRs and were kept in white .
