# Supplementary material for: Qualitative and quantitative assessment of Illumina’s forensic STR and SNP kits on MiSeq FGx™
Source: PLoS One. 2017 Nov 9;12(11):e0187932. doi: 10.1371/journal.pone.0187932 (PMC5679668; doi:10.1371/journal.pone.0187932)
Supplement: S4 Fig — (PDF) [file pone.0187932.s004.pdf]

Suppl. Fig. 4

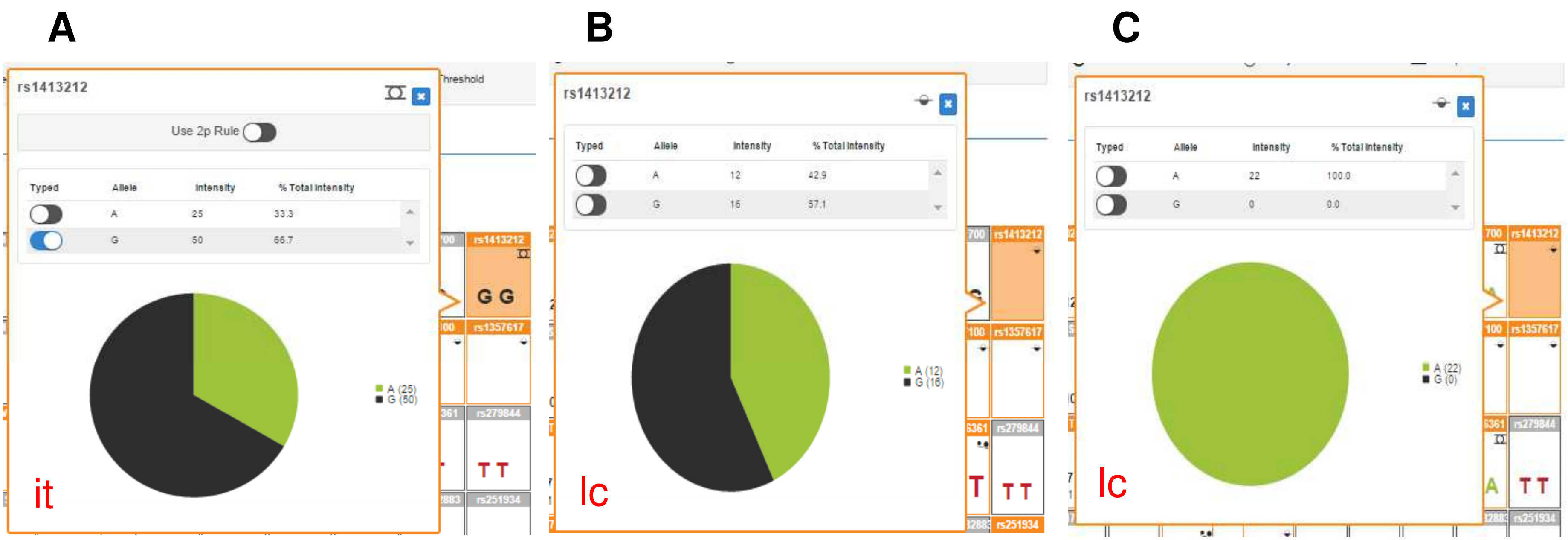

UAS data output for iSNPs

Three UAS outputs from the same individual at rs1413212 depending on read numbers. **A**. The locus was flagged with interpretation threshold (it). Only allele G was typed, even though allele A also contained some reads (25). **B**. The locus was flagged with low coverage (lc) and no allele was typed despite the fact that both contained some reads (G: 16 and A: 12). **C**. The locus was flagged with low coverage (lc) and no allele was typed. Only one allele contained some reads (G: 0 and A: 22).
