# Supplementary material for: Qualitative and quantitative assessment of Illumina’s forensic STR and SNP kits on MiSeq FGx™
Source: PLoS One. 2017 Nov 9;12(11):e0187932. doi: 10.1371/journal.pone.0187932 (PMC5679668; doi:10.1371/journal.pone.0187932)
Supplement: S1 Table — Typed genotype differs from reference sample. (PDF) [file pone.0187932.s005.pdf]

Suppl. Table 1: Genotype errors not flagged, Fig. 2 marked in red (n=71). Typed genotype differs from reference sample

Expt.	Locus	N	Genotype	Reference	Comment
II	vWA	1	16	16,17	ADO: typed as homozygote
	PentaD	2	9; 13	9,15; 13,16	2x ADO: typed as homozygote
	DYS385a-b	3	11; 11; 10,11	11,19; 11,13; 11,14	2x ADO: typed as homozygote; ADO and typed stutter
	DXS10103	1	20	19,20	ADO: typed as homozygote
	DXS10135	1	20.1	20.1,33	ADO: typed as homozygote
III	DYS385a-b	3	11; 11; 13	11,19; 11,19; 12,13	3x ADO: typed as homozygote
IV	DYS385a-b	1	11	11,19	ADO: typed as homozygote
V	D1S1656	1	16	15,16	ADO: typed as homozygote
	PentaE	1	12	12,19	ADO: typed as homozygote
	DXS10103	2	15,18;18	16,18; 16,18	ADO and typed stutter; ADO: typed as homozygote
VI	CSF1PO	1	12	10,12	ADO: typed as homozygote
	D18S51	1	15	14,15	ADO: typed as homozygote
	D1S1656	1	15	15,16	ADO: typed as homozygote
	D10S1248	1	15	15,16	ADO: typed as homozygote
	DXS10103	2	18;16	16,18; 16,18	2x ADO: typed as homozygote
VII	D7S820	1	13	8,13	ADO: typed as homozygote
	D18S51	1	16	15,16	ADO: typed as homozygote
	D12S391	1	20	19,20	ADO: typed as homozygote
	PentaE	1	20	16,20	ADO: typed as homozygote
	D17S1301	1	12	12,13	ADO: typed as homozygote
	PentaD	4	11; 9; 11; 9	11,13; 9,13; 11,13; 9,13	4x ADO: typed as homozygote
	DYF387S1	1	37	37,41	ADO: typed as homozygote
	DXS10074	1	17	16,17	ADO: typed as homozygote
VIII	CSF1PO	1	9	9,11	ADO: typed as homozygote
	vWA	1	16	14,16	ADO: typed as homozygote
	PentaD	12	9; 9; 10; 10; 11; 11; 9; 10; 10; 9; 9; 9	9,13; 9,13; 10,12; 10,12; 11,13; 11,13; 9,12; 10,13; 10,13; 9,15; 9,13; 9,13	12x ADO: typed as homozygote
	DYF387S1	2	36; 37,38	36,38; 38,39	ADO: typed as homozygote; ADO and typed stutter
	DYS448	1	7.2	22	LDO and ADI (# of reads: 12) typed
	DXS10103	3	18; 20; 19	16,18; 16,20; 16,19	3x ADO: typed as homozygote
	DXS10135	5	24; 24; 20.1; 20.1; 20	24,28; 24,28; 20.1,33; 20.1,33; 20,25	5x ADO: typed as homozygote
IX	FGA	3	18; 23; 20	18,26; 23,24; 20,26	3x ADO: typed as homozygote
	D7S820	1	8	8,12	ADO: typed as homozygote
	D12S391	3	18; 20; 20	18,20; 20,22; 20,23	3x ADO: typed as homozygote
	D16S539	1	11	10,11	ADO: typed as homozygote
	DXS10103	5	18; 16; 16; 20; 19	16,18; 16,18; 16,20 ; 19,20; 19,19	5x ADO: typed as homozygote
