# Supplementary material for: Qualitative and quantitative assessment of Illumina’s forensic STR and SNP kits on MiSeq FGx™
Source: PLoS One. 2017 Nov 9;12(11):e0187932. doi: 10.1371/journal.pone.0187932 (PMC5679668; doi:10.1371/journal.pone.0187932)
Supplement: S2 Table — (PDF) [file pone.0187932.s006.pdf]

Suppl. Table 2: Genotype errors flagged, Fig. 2 marked in orange (n=8)

Expt.	Sample	Locus	Flag[1]	Genotype typed	Reference Genotype	Error	Comment
I	B7Fb	DYS392	ma	11	N/A	Female sample	# of reads: 13
II	B8Ma	DYS385a-b	i	10,11	11,13	Typed stutter, ADO	Genotype error
II	B9Ma	DYS385a-b	i	9,10	10,14	Typed stutter, ADO	Genotype error
II	B9Mb	DYS385a-b	i	9,10	10,14	Typed stutter, ADO	Genotype error
V	B7F_800	DYS505	ma	13	N/A	Female sample	# of reads: 16
VI	B5F_400	DYS576	ma	16	N/A	Female sample	# of reads: 60
IX	B14F	DYS643	ma	10	N/A	Female sample	# of reads: 37
IX	B8M	D17S1301	i	10,12	11,12	Typed stutter, ADO	Genotype error

[1]**Flag:** ma: many alleles/allele count; i: imbalance
