# Supplementary material for: Qualitative and quantitative assessment of Illumina’s forensic STR and SNP kits on MiSeq FGx™
Source: PLoS One. 2017 Nov 9;12(11):e0187932. doi: 10.1371/journal.pone.0187932 (PMC5679668; doi:10.1371/journal.pone.0187932)
Supplement: S3 Table — (PDF) [file pone.0187932.s007.pdf]

Suppl. Table 3: Assessing cross-contamination by mixing samples directly after indexing and after PCR purification

Mixed DNA sample	GE	Concordance				Typed ADI				Untyped ADI				ACR			
		aSTR	Y-STR	X-STR	iSNP	aSTR	Y-STR	X-STR	iSNP	aSTR	Y-STR	X-STR	iSNP	aSTR	Y-STR	X-STR	iSNP
After indexing: F	0	100%	N/A	100%	100%	0	0	0	0	<1%	0	<1%	0	1	N/A	0	3
After indexing: M	0	100%	100 %	100%	100%	0	0	0	0	<1%	<3%	<1%	1	3	0	N/A	3
After PCR purification: F	0	100%	N/A	100%	100%	0	0	0	0	<1%	0	<1%	0	2	N/A	1	1
After PCR purification: M	0	100%	100 %	100%	100%	0	0	0	0	<1%	<3%	<1%	0	2	0	N/A	1

GE (Genotype Error): 0: no genotype error was detected.

Concordance: The four samples showed the same genotypes as in Expt. I, when these samples were used as single-source samples.

Typed ADI: 0: no typed ADI was found.

Untyped ADI: For STRs the percentage to the true allele is given and for iSNPs, the number of cases is given. Y-STRs: female samples showed no untyped ADI. The untyped ADI of male samples were <3%. Only one iSNP (rs6955448) showed a few untyped reads (12) of the other allele (T). The true allele C had 2381 reads (ACR: 0.005) and the genotype was given by the UAS as homozygous CC.

ACR: The number of loci that had ACRs <0.6 is given. For STRs, besides D22S1045, the ACRs were >0.5, while for D22S1045, the ACRs were as low as 0.27. For iSNPs, besides the ACR at rs6955448, which had an ACR of 0.005 (male after indexing), the other ACRs ranged from 0.42 – 0.60.
