# Supplementary material for: Qualitative and quantitative assessment of Illumina’s forensic STR and SNP kits on MiSeq FGx™
Source: PLoS One. 2017 Nov 9;12(11):e0187932. doi: 10.1371/journal.pone.0187932 (PMC5679668; doi:10.1371/journal.pone.0187932)
Supplement: S4 Table — (PDF) [file pone.0187932.s008.pdf]

Suppl. Table 4: Typed stutter and typed sequence error, Fig. 2 marked in pink (n=18)

Expt.	Sample	Locus	DNA Input [pg]	Genotype typed	Reference Genotype	Comment and % of True allele
I	BM1a	D7S820	1000	**10**, 10.1, 11	10	Typed (+1) stutter 1%, ADI: Insertion of one T (3%)
	B4Mb	D7S820		**10**, 10.1, 11, **12**, 12.1	10,12	Typed (±1) stutter 11%, 2 ADI: Insertion of one T (<4%)
	B5Fa	D7S820		**8**, 8.1, **12**, 12.1, 13	8,12	Typed (+1) stutter 2%, 2 ADI: Insertion of one T (<3%)
	B5Fb	D7S820		**8**, 8.1, **12**, 12.1, 13	8,12	Typed (+1) stutter 1%, 2 ADI: Insertion of one T (<3%)
	B6Fa	D7S820		**10**, 10.1, **11**, 11.1, 12	10,11	Typed (+1) stutter 2%, 2 ADI: Insertion of one T (<2%)
	B7Fa	D7S820		**8**, 8.1, **13**, 13.1, 14	8,13	Typed (+1) stutter 2%, 2 ADI: Insertion of one T (<2%)
	B7Fb	D7S820		**8**, 8.1, **13**, 13.1, 14	8,13	Typed (+1) stutter 1%, 2 ADI: Insertion of one T (<2%)
	B13Fb	D7S820		**10**, **11**, 11.1, 12	10,11	Typed (+1) stutter 2%, ADI: Insertion of one T (2%)
	B15Fa	D7S820		**10**, 10.1, **11**, 11.1, 12	10,11	Typed (+1) stutter 1%, 2 ADI: Insertion of one T (<3%)
	B16Fa	D7S820		**9**, 9.1, 10, **12**, 12.1	9,12	Typed (+1) stutter 1%, 2 ADI: Insertion of one T (<3%)
	B16Fb	D7S820		**9**, 10, **12**, 12.1	9,12	Typed (+1) stutter 2%, ADI: Insertion of one T (2%)
II	B8Ma	D7S820	1000	**8**, 8.1, **12**, 13	8,12	Typed (+1) stutter 2%, ADI: Insertion of one T (3%)
	B1Ma	DYS390		23, **24**, 24, 24	24	Typed (-1) stutter 18%, 2 ADI: Substitution of two nt (<8%)
	B10Ma	DYS390		24, **25**, 25	25	*Genotype determined on read #s:* Typed (-1) stutter 26%, ADI: Substitution of one nt (22%)
VII	B1M	DYF387S1	800	**36**, 37, **38**, 38	36,38	Typed (±1) stutter 24%, ADI: Substitution of one nt (22%)
VIII	B10Ma	DYF387S1	500	36, **37**, 37, 38, 39, 39, **40**, 40, 40, 40, 40	37,40	*Genotype determined on read #s*: 2 typed (-1 and+1) stutter, 6 ADI: Substitution of one nt (25-43%), Substitution of two nt (4.8 and 40%)
IX	B9M	D7S820	100	9, **10**, **12**, 12	10,12	Typed (-1) stutter 10%, ADI: Substitution of one nt (44%)
	B9M	D6S1043		**11**, 18, **19**, 19	11,19	Typed (-1) stutter 13%, ADI: Substitution of one nt (22%)

nt: nucleotide
