# Supplementary material for: Qualitative and quantitative assessment of Illumina’s forensic STR and SNP kits on MiSeq FGx™
Source: PLoS One. 2017 Nov 9;12(11):e0187932. doi: 10.1371/journal.pone.0187932 (PMC5679668; doi:10.1371/journal.pone.0187932)
Supplement: S5 Table — (PDF) [file pone.0187932.s009.pdf]

Suppl. Table 5: Typed sequence errors, Fig. 2 marked in purple (n=203)

Expt.	STRs	Locus	DNA Input [pg]	N	% of true allele	Comment
I	a-STR	D7S820	1000	17	<3	Insertion of one T at the end of the sequence
	Y-STRs	DYS643		3	1%	Deletion of one T in first repeat
II	a-STRs	D7S820	1000	12	1.3-3.2	D7S820: Insertion of one T at the end of the sequence
		FGA		1	<1	FGA:Deletion of one T in last repeat
	Y-STRs	DYS643		2	<1	Deletion of one T
		DYS448		1	<20	Substitution of one nt
		DYS390		7	5-25	Substitution of one (21-25 %) or two nt (5-8.8 %)
IV	a-STRs	D7S820	1000	53	<5	Insertion of one T at the end of the sequence
V	a-STRs	D7S820	800-400	7	<3	Insertion of one T at the end of the sequence
		D1S1656	100-50	2	33-50	Substitution of one nt
		D4S2408	50	1	25	Substitution of one nt
	Y-STRs	DYS576	800	1	<0.5	Deletion of one A
		DYS643	800, 100	3	0.5-1.7	Deletion of one T
		DYS437	50	1	25	Substitution of one nt
	X-STRs	DXS10135	100	1	3	Substitution of one nt and deletion of one nt
VI	a-STRs	D7S820	800-100	7	≤4	Insertion of one T
		D19S433	400	1	1.5	Substitution of one nt and insertion of two A
	X-STRs	DXS7132	800-200	3	<1	Insertion of one T
VII	Y-STRs	DYS385a-b	800	1	≤56	Substitution of one nt
		DYS390	800-400	2	20-23	Substitution of one nt
VIII	a-STRs	D7S820	500	1	3	Insertion of one T at the end of the sequence
				1	22	Substitution of one nt
	Y-STRs	DYS643		1	0.4	Deletion of one nt
		DYF387S1		1	1.6-2.5	Substitution of three (1.6-2.5 %) or four nt (1.6 %)
	X-STRs	DXS7132		20	<5	Insertion of one T
IX	a-STRs	D1S1656	100	7	16-37	Substitution of one nt (27-37 %) or two nt (16 %)
		D7S820		6	22-48	Substitution of one nt
		D12S391		4	8-34	Substitution of one nt
		FGA		3	8-26	Substitution of one nt
		D6S1043		1	28	Substitution of one nt
	Y-STRs	DYS389II		4	27-52	Substitution of one nt
		DYS460		4	13-27	Substitution of one (21-27 %) or two nt (13 %)
	X-STRs	DXS7132		13	5-14	Insertion of one nt
		DXS10074		11	1-82	Substitution of one nt (20-82 % of true allele), Substitution of two nt (5-38% of true allele), Substitution of three nt (4-10% of true allele), Substitution of four nt (1-3.5% of true allele)

N: number of occurrences
nt: nucleotide
