# Supplementary material for: Qualitative and quantitative assessment of Illumina’s forensic STR and SNP kits on MiSeq FGx™
Source: PLoS One. 2017 Nov 9;12(11):e0187932. doi: 10.1371/journal.pone.0187932 (PMC5679668; doi:10.1371/journal.pone.0187932)
Supplement: S6 Table — (PDF) [file pone.0187932.s010.pdf]

Suppl. Table 6: Untyped sequence errors at **D19S433** from one reaction sample of Expt. I

Allele Name	Typed Allele?	Reads	Repeat Sequence	Comments
14	No	100	AAGG AAAG AAGG TAGG AAGG AAGG AAGG AAGG AAGG AAGG AAGG AAGG AAGG AAGG AAGG AAGG AGAG AGGA AGAA AGAGAG	(-1) Stutter
15	No	16	AAGG AAAG AAGG TAGG **T**AGG AAGG AAGG AAGG AAGG AAGG AAGG AAGG AAGG AAGG AAGG AAGG AAGG AGAG AGGA AGAA AGAGAG	Substitution of one nt
15	No	14	AAGG AAAG AAGG TAGG AAGG AAGG AAGG AAGG AAGG AAGG AAGG AAGG AAGG AAGG AAGG AAGG AAGG AGAG AG**A**A AGAA AGAGAG	Substitution of one nt
15	No	12	AAGG AAAG AAGG TAGG AAGG AAGG AAGG AAGG AAGG AAGG AAGG AAGG AAGG AAGG AAGG AAGG AAGG AGAG A**A**GA AGAA AGAGAG	Substitution of one nt
15	Yes	1436	AAGG AAAG AAGG TAGG AAGG AAGG AAGG AAGG AAGG AAGG AAGG AAGG AAGG AAGG AAGG AAGG AAGG AGAG AGGA AGAA AGAGAG	**True Allele**
16.2	No	104	AAGG AA AAGG TAGG AAGG AAGG AAGG AAGG AAGG AAGG AAGG AAGG AAGG AAGG AAGG AAGG AAGG AAGG AAGG AGAG AGGA AGAA AGAGAG	(-1) Stutter
17.2	No	14	AAGG AA AAGG TAGG AAGG AAGG AAGG AAGG AAGG AAG**A** AAGG AAGG AAGG AAGG AAGG AAGG AAGG AAGG AAGG AAGG AGAG AGGA AGAA AGAGAG	Substitution of one nt
17.2	No	29	AAGG AA AAGG TAGG AAGG AAGG AAGG AAGG AAGG AA**A**G AAGG AAGG AAGG AAGG AAGG AAGG AAGG AAGG AAGG AAGG AGAG AGGA AGAA AGAGAG	Substitution of one nt
17.2	No	28	AAGG AA AAGG **A**AGG AAGG AAGG AAGG AAGG AAGG AAGG AAGG AAGG AAGG AAGG AAGG AAGG AAGG AAGG AAGG AAGG AGAG AGGA AGAA AGAGAG	Substitution of one nt
17.2	No	27	AAGG AA AAGG **G**AGG AAGG AAGG AAGG AAGG AAGG AAGG AAGG AAGG AAGG AAGG AAGG AAGG AAGG AAGG AAGG AAGG AGAG AGGA AGAA AGAGAG	Substitution of one nt
17.2	No	21	AA**A**G AA AAGG TAGG AAGG AAGG AAGG AAGG AAGG AAGG AAGG AAGG AAGG AAGG AAGG AAGG AAGG AAGG AAGG AAGG AGAG AGGA AGAA AGAGAG	Substitution of one nt
17.2	No	13	AAGG AA AAGG TAGG AAGG AAGG AAGG AAGG AA**A**G AAGG AAGG AAGG AAGG AAGG AAGG AAGG AAGG AAGG AAGG AAGG AGAG AGGA AGAA AGAGAG	Substitution of one nt
17.2	Yes	894	AAGG AA AAGG TAGG AAGG AAGG AAGG AAGG AAGG AAGG AAGG AAGG AAGG AAGG AAGG AAGG AAGG AAGG AAGG AAGG AGAG AGGA AGAA AGAGAG	**True Allele**
